# Simulated blast overpressure induces specific astrocyte injury in an *ex vivo* brain slice model

**DOI:** 10.1371/journal.pone.0175396

**Published:** 2017-04-12

**Authors:** Saranya Canchi, Malisa Sarntinoranont, Yu Hong, Jeremy J. Flint, Ghatu Subhash, Michael A. King

**Affiliations:** 1 Department of Mechanical & Aerospace Engineering, University of Florida, Gainesville, Florida, United States of America; 2 Mcknight Brain Institute, University of Florida, Gainesville, Florida, United States of America; 3 Department of Pharmacology & Therapeutics, University of Florida, Gainesville, Florida, United States of America; 4 North Florida/South Georgia VA Medical Center, Gainesville, Florida, United States of America; Fraunhofer Research Institution of Marine Biotechnology, GERMANY

## Abstract

Exposure to explosive blasts can produce functional debilitation in the absence of brain pathology detectable at the scale of current diagnostic imaging. Transient (ms) overpressure components of the primary blast wave are considered to be potentially damaging to the brain. Astrocytes participate in neuronal metabolic maintenance, blood–brain barrier, regulation of homeostatic environment, and tissue remodeling. Damage to astrocytes via direct physical forces has the potential to disrupt local and global functioning of neuronal tissue. Using an *ex vivo* brain slice model, we tested the hypothesis that viable astrocytes within the slice could be injured simply by transit of a single blast wave consisting of overpressure alone. A polymer split Hopkinson pressure bar (PSHPB) system was adapted to impart a single positive pressure transient with a comparable magnitude to those that might be present inside the head. A custom built test chamber housing the brain tissue slice incorporated revised design elements to reduce fluid space and promote transit of a uniform planar waveform. Confocal microscopy, stereology, and morphometry of glial fibrillary acidic protein (GFAP) immunoreactivity revealed that two distinct astrocyte injury profiles were identified across a 4 hr post-test survival interval: (a) presumed conventional astrogliosis characterized by enhanced GFAP immunofluorescence intensity without significant change in tissue area fraction and (b) a process comparable to clasmatodendrosis, an autophagic degradation of distal processes that has not been previously associated with blast induced neurotrauma. Analysis of astrocyte branching revealed early, sustained, and progressive differences distinct from the effects of slice incubation absent overpressure testing. Astrocyte vulnerability to overpressure transients indicates a potential for significant involvement in brain blast pathology and emergent dysfunction. The testing platform can isolate overpressure injury phenomena to provide novel insight on physical and biological mechanisms.

## Introduction

Blast induced traumatic brain injury (bTBI) is a debilitating condition that can alter the neurological function of an affected individual [1]. A primary blast wave is characterized by a rapid rise in pressure sometimes followed by a negative pressure. Propagation produces transient, localized compressive followed by tensile stress over a duration that may be less than a millisecond [2]. In heterogeneous materials characterized by different impedances, such high-rate strains can cause structural failures not observed following slower distorting events. Differential energy dissipation at tissue interfaces can produce rapid directional shear, tension and compression sufficient to cause fracture in rigid elements, and tearing of soft tissues. The particular susceptibility of long tubular structures to such mechanical failures manifests in the brain as vascular rupture and breakage of axons [2–4].

Blast TBI neuropathology is often undetectable with *in vivo* diagnostic imaging, so the structural basis of behavioral, intellectual and physiological deficits in blast survivors is likely due to injuries at a finer spatial scale than present technology can resolve [5]. Recently diffusion tensor imaging (DTI) was shown to capture axonal injury in military personnel suffering from bTBI [6]. However, contemporary studies have not yet resolved whether brain tissue can be directly damaged by blast waves or the extent to which this combines with skull contact (coup/contrecoup), hydraulic and pneumatic effects (vascular, lung, sinus), or other sources of injury. The presumption that direct neuronal susceptibility underlies bTBI neuropathology is predicated on an absence of study of blast effects on other brain cells.

Astrocytes, a subtype of glial cells, play pivotal roles in brain homeostasis, neuronal metabolism, and synaptic signaling [7, 8]. They have long been known to participate in protracted tissue remodeling that occurs in response to injury, but their properties suggest multiple ways they may be involved in influencing neurons or other cells. Astrocytes possess unique and spatially complex morphological structure including elaborate tubular branches that extend from astrocyte perikarya, and fine sheet processes in distal arbors. Their fine processes compartmentalize blood vessels and synaptic terminals, and mediate both physical and chemical functions within these specialized structures [9–11]. Such structures could be particularly susceptible to damage from transient forces occurring during a TBI event. Furthermore, because each astrocyte defines a distinct, non-overlapping, and stable tissue compartment within the extent of its arborized processes [12], injury to an individual astrocyte could compromise the function of multiple neurons with axons or dendrites supported in these volumes. The multiple branched and interdigitated processes of astrocytes and neurons suggest that vulnerability to deformation injuries is likely to be shared by both cell types. Activation of astrocytes measured by glial fibrillary acidic protein (GFAP) has been previously noted following long time periods (>2 days) after *in vivo* exposure to blast waves characterized by overpressure and underpressure components [1, 13, 14]. Delayed response (>1 day) of injured astrocytes in culture shows changes in survival and reactivity gene expression [15] with decreased adenosine triphosphate (ATP) levels, along with increases in both reactive oxygen species (ROS) formation and cellular injury [16]. The possibility of more rapid reactivity is suggested by transient upregulation of immediate early genes (IEGs) has been reported to peak 30–60 min after mechanical injury, and eventually subsides after 3 hrs in cultured glial cells [17]. However the acute response of astrocytes *in situ* to transient pressure gradients have received little study.

Experimental study of bTBI can be improved by simplifying the variable features involved in testing. *In vivo* models that have looked at primary blast wave effects frequently use shock tubes. A wide range of pathologies have been reported based on the peak magnitude of pressure profile used [1, 18], orientation of the head with respect to the shock tube axis [19], anesthesia [20, 21], the presence or absence of protective wear [18, 22], placement i.e. inside vs outside in relation to the shock tube [14, 23] and size of the animal with respect to the tube diameter [24, 25]. Although *in vivo* models allow for study of physiological and neurobehavioral response to blast waves, injury to the brain is often confounded with injuries resulting from brain and skull impedance mismatch [26, 27], skull flexure [28] and pressure gradients developing between coup and countrecoup regions [29]. Limited visual accessibility, lack of spatial and temporal resolution of tissue strain history, and associated time and cost with animal care are all limitations of *in vivo* models, and thus make *in vitro* models a useful complementary approach [3].

To investigate brain tissue-specific vulnerability at relevant spatial and temporal scales, we have developed a viable brain slice model that isolates blast wave effects at cellular and local-circuit scales from those associated with skull (flexure effects and coup/contrecoup), vasculature (thoracic compression waves, microbleeds, and embolism) and organ-scales (long axonal pathway tension and torsion) [30]. The test system utilizes a polymer split Hopkinson pressure bar (PSHPB) [31] to expose the soft tissue sample to high strain rate loading that simulates components of blast. The pressure waves generated from this system approximated relevant blast wave characteristics with excellent control and reproducibility. The induced tissue deformation was non-uniform, with mixed modes of strain including tension, compression and shear. Characterization of the mechanical properties and viability of neuronal populations over 8 hr slice incubations established effects of shock beyond those associated with the slice procedure [32, 33]. The multiple branched and interdigitated processes of astrocytes and neurons suggest that vulnerability to deformation injuries is likely to be shared by both cell types.

For the present study, a modified design for the PSHPB test system incorporated a more compact, rectangular test chamber to propagate a single positive pressure transient with a comparable magnitude to those that might be transmitted into the head. Our test paradigm allows us to examine the hypothesis that injury to astrocytes within the slice can occur due to overpressure alone (i.e. without negative pressure components). This ability is nontrivial because overpressure magnitude has been established previously in the literature as a damage-inducing factor, and underpressure may have unique contributions [34].

## Materials and methods

### Acute brain tissue slice

Protocols and procedures for this study conform to NIH guidelines and were approved by the University of Florida Institutional Animal Care and Use Committee. The acute *ex vivo* slice model which conserves the native tissue architecture was refined from previous work [30]. Young adult male Sprague-Dawley rats (Harlan) were experimental subjects on the basis of an extensive brain slice literature and neurobiological similarities with humans. On each experiment day, one rat (~ 3 months old) was deeply anesthetized by inhalation of the short-acting volatile anesthetic isoflurane (5%), decapitated and the extracted intact brain was submerged in ice-cold artificial cerebrospinal fluid (aCSF) containing 120 mM NaCl, 3 mM KCl, 2 mM CaCl_2_.2H_2_O_,_ 1.4 mM MgSO_4_.7H_2_O_,_ 26 mM NaHCO_3,_ 1.5 mM KH_2_PO_4,_ and 10 mM glucose; 300 ± 2 mOsm and saturated with carbogen gas (95% O_2_ + 5% CO_2_; pH 7.3–7.4). Coronal tissue slices (300 μm) of the forebrain were obtained using a Vibratome (Leica VT 1000A, Leica Microsystems Inc., Germany) and transferred to a holding chamber containing aCSF (35–37°C) that was continuously perfused with carbogen gas. Subsequently, an individual slice was transferred to the test chamber filled with carbogen saturated aCSF (35–37°C) for the duration of the test before being returned to the holding chamber for varying incubation times.

### Overpressure load testing

**Fig 1:**
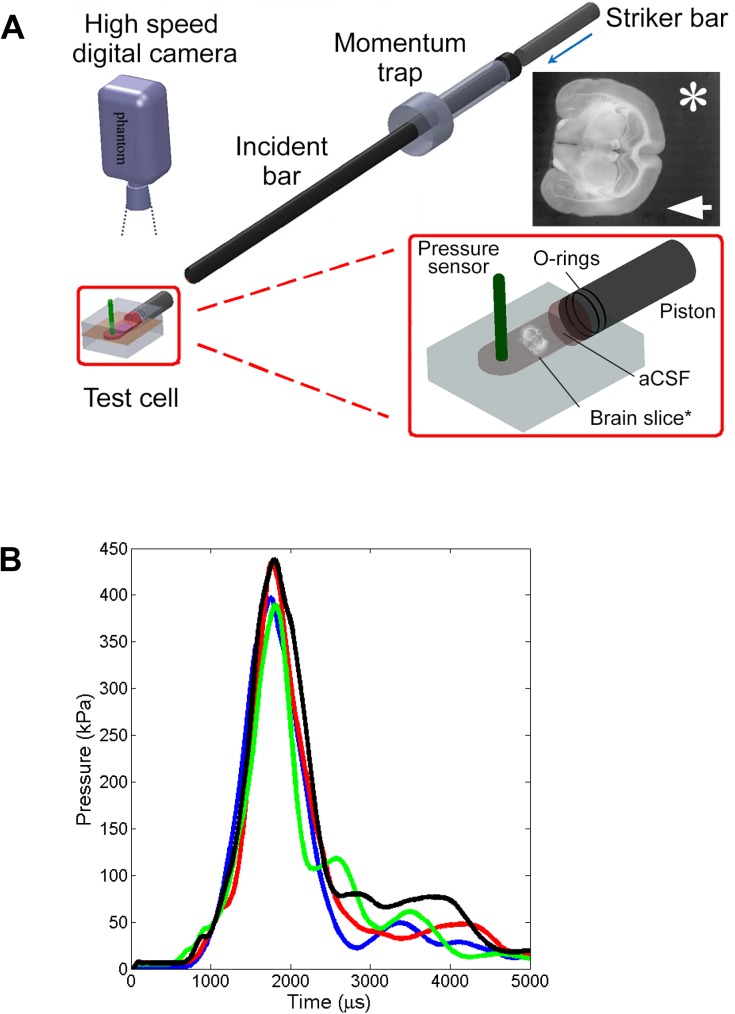
Schematic of the PSHPB system with the custom transparent test cell. (A) Schematic shows the test cell integrated with the PSHPB apparatus (Length = 2.52 m, Diameter = 25.4 mm). Insert enlargement shows the positioning of the brain tissue slice in the interior chamber, suspended in carbogen-saturated aCSF. The pressure sensor recorded the pressure inside the chamber during testing. A high speed camera was focused on the brain slice to capture slice deformation. * Indicates video frame of the brain tissue slice within the chamber during testing. The direction of the simulated overpressure wave was from right to left (arrow). The distance between the slice and the piston was > 1 cm. Inherent contrast of the tissue is sufficient to identify internal structural features of the brain. (B) Representative test chamber pressure profiles from 4 tests illustrate the consistent overpressure magnitude and duration, and absence of underpressure in the simulated blast wave. Average peak pressure inside the chamber was 60.25 ± 4.99 psi (415.40 ± 34.40 kPa). The average full width at half maximum was 362 μs. The system allowed for a single peak loading along with absence of underpressure (negative pressure component).

Experiments were designed to determine the evolution of injury of live embedded cells in response to a single overpressure exposure in absence of other compounding biomechanical loading for varying post-test incubation times (0–4 hrs). The magnitude of the overpressure measured within aCSF fluid chamber was on par with pressure measurements taken in animal CSF spaces in prior field tests [25, 35, 36]. Coronal slices between the Paxinos and Watson rat brain atlas Bregma levels -2.30 to -3.80 mm were selected for testing [37]. To introduce a planar pressure wave to the sealed fluid test chamber containing brain tissue slices, a customized polymer split Hopkinson pressure bar (PSHPB) test system was adapted (Fig 1A) [30]. A nitrogen gas gun pressure of 10 psi (~69 kPa) controlled the velocity of a striker bar, and was constant for all experiments. Impact of the high velocity striker bar with an incident bar imparted a stress wave to travel into the custom built test cell. Regional variation in tissue mechanical properties (effective modulus) along with viscoelastic property of brain tissue dictate that for exposure to approximately planar stress/pressure results in non-homogenous strains and stresses[26, 32] which is applicable to this study. Non-uniform strains are also expected due to transmission of the stress wave itself. In-plane strains due to the overpressure exposure were not measured due to the resolution limit of the current imaging set up (< 0.002, detectable strain limit and minimal displacement during overpressure) using image analysis methods as described in our previous studies [30]. Thus, the reasonable measured pressure profiles within the chamber are indicative of the stress wave transmitted to the tissue slice.

The 90 mm x 55 mm x 42 mm transparent acrylic test cell (Fig 1A insert) consisted of a cylindrical hole through which a piston was fit connected to a fluid-filled, interior rectangular chamber [50 mm x 25 mm x 4 mm] to house the brain slice. The fluid chamber was machined to provide a leak-proof interface with the piston rod. Immediately (ca. 1 min) prior to each experiment, the test cell was filled with 6 ml of carbogen-saturated aCSF (35–37°C). A single tissue slice was approximately centered in the chamber using a spatula, but not secured physically to any surface. After slice placement, the piston rod outfitted with rubber O-rings and lubricated with pharmaceutical grade petroleum jelly to avoid leakage of aCSF was slid into place and the assembled test chamber integrated with the PSHPB apparatus.

With PSHPB testing, stress wave transmission through the piston assembly generated an overpressure wave in the test chamber fluid (Fig 1A) but no other action (e.g. physical contact) on the brain slice. Refinements from previous designs include rectangular chamber geometry devised to support planar wave transit, and reduced fluid volume to restrict tissue displacement [30]. Pressure data were collected at 100 kHz (LDS Nicolet, Sigma 90) through a high rate dynamic pressure sensor (PCB Piezotronics, #113B24) fitted in the top wall of the test cell. A high speed camera (Vision Research, Wayne, NJ, 100,000 frames/second) positioned above the test chamber captured tissue deformation. Images were captured at a resolution of 608 x 512 pixels (field of view of 12.1mm x 10.2mm), at a sample rate of 21,000 fps. The PSHPB system also incorporated a momentum trap, consisting of a sleeve at the end of the incident bar, to impart a controlled, single stress wave and minimize low amplitude rebound loading. A more detailed description of this system’s operation can be found in Subhash and Ravichandran [38].

Individual tests were completed in less than 5 min and consisted of exposure to one applied overpressure wave in the fluid chamber containing one slice. Throughout the duration of the test, the piston remained in contact with the aCSF; the brain slice experienced no physical contact apart from resting on the chamber floor. For controls, single slices were introduced into the test cell along with aCSF and held in chamber for the typical test duration time (4–5 min) without exposure to a pressure wave. Upon test completion, the test chamber was disassembled, and brain tissue slices were carefully returned to the holding chamber for continued incubation in 35–37°C carbogen perfused aCSF. To test the changes within functional astrocytes, the incubation time points tested were 0, 0.5, 1, 2, 3 and 4 hours post testing. Treatment condition (control or overpressure-exposed) and the incubation duration were distributed randomly over the 26 included slices obtained from 6 rats.

### GFAP immunohistology

After concluding post-test incubation, brain tissue slices were immersion-fixed for 2 hours in 4% formaldehyde in 0.1 M sodium phosphate buffer solution (PBS) pH 7.2–7.4, rinsed for 15 minutes in PBS and stored in fresh PBS. To immunolabel astrocytes, whole slices were incubated with a monoclonal antibody against GFAP (1:250, Sigma Aldrich or BD Bioscience) for 24 hours at room temperature, followed by anti-mouse immunoglobulin conjugated to fluorescent label Alexa 488 (1:500, Life Technologies), before being mounted on slides and coverslipped with glycerol gelatin.

### Confocal imaging

Examination was restricted to the stratum radiatum of the CA1 hippocampal subregion. Hippocampal injury and associated functional deficits are prevalent in blast TBI [13, 14, 21, 22], and CA1 stratum radiatum provides a relatively homogenous sampling compartment. Hippocampi were imaged bilaterally in all slices across all incubation times and overpressure conditions. In randomly selected microscope fields, beginning 15 μm below the slice surface, three-dimensional stacks of 1024 x 1024 pixel 8 bit intensity channel images were acquired from intact GFAP-immuno-labeled slices using a Leica SP2 confocal microscope (Cell & Tissue Analysis Core, McKnight Brain Institute, University of Florida). A water immersion (63x) objective was used to collect photoemission from slices illuminated by a helium-neon laser source (488 nm). Gain, offset, aperture and laser power were kept constant between images and slices. Z-stacks were collected at 1 μm intervals as deep as permitted by the objective working distance (~ 220 μm) and antibody penetration.

### Quantification of data

To identify and assess astrocyte integrity, and area fraction of GFAP immunoreactivity, the average intensities of GFAP-positive pixels were quantified and analyzed for all images across treatment groups. Area fraction of the GFAP immunoreactivity was first calculated and used to determine the average fluorescence intensity within GFAP-positive pixels. Using Fiji image processing software [39], maximum-intensity z-projection images were obtained from each 3D stack of confocal images. After inverting the intensity to generate white backgrounds, images were thresholded interactively to include the smallest GFAP-immunopositive features and mask pixels positive for GFAP immunoreactivity. Masks were overlaid on the z-projection image to calculate the immunoreactive area fraction (ratio of thresholded labeled pixels to the total number of image pixels in a given image field) and the average intensity (sum of all GFAP+ pixel intensities divided by the number of pixels within the mask).

Area fraction and average GFAP intensity were averaged across image fields, brain hemisphere and slices to generate within-animal mean values for a given time and treatment condition. In reviewing immunolabeled samples we observed multiple GFAP-positive cells that contained abnormal intracellular regions devoid of GFAP immunoreactivity that were never seen in control cells. The ratio of such cells to the total number of labeled astrocytes was estimated from summary counts of cells with and without such intracellular regions. The number of GFAP labeled astrocytes for a given treatment and incubation time was manually counted based on visual inspection of the maximum intensity z-projection images. The total number of astrocytes averaged across all image fields for a given treatment at a given incubation time provided an index of the number of affected astrocytes per image field.

### Quantitative morphology assessment

**Fig 2:**
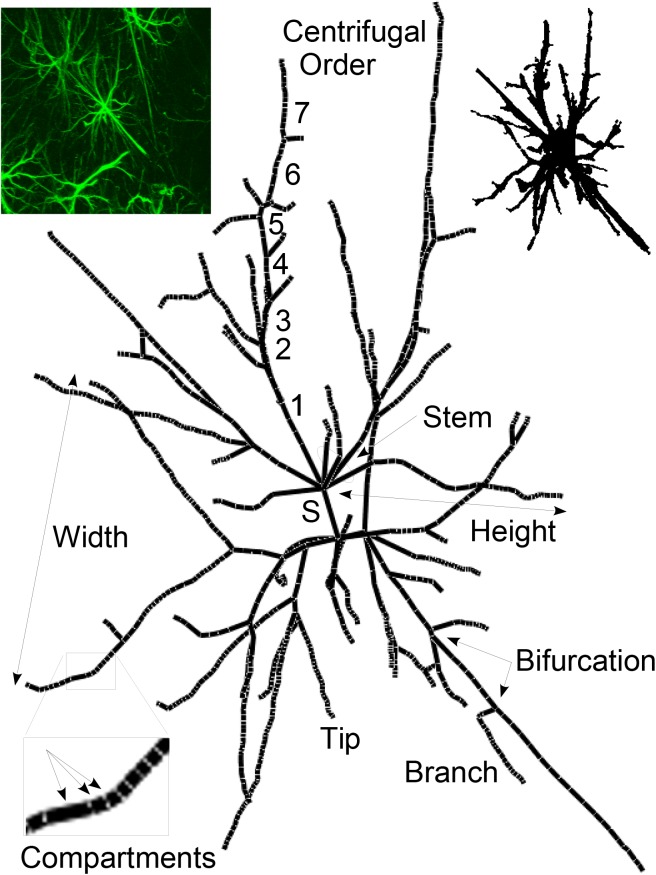
Elementary topological properties for an astrocyte. A sub-set of measurements obtained in this study are highlighted. The complete list of graphic descriptors of L-Measure is available at http://cng.gmu.edu:8080/Lm/help/index.htm [41]. The primary processes attached to the soma (Stems) are comprised of branches, terminal segments and terminal tips. Branches form the continuous segment between bifurcation points and are made up of one or more cylindrical compartments generated by the NeuronStudio tracing algorithm. The branching order was centrifugal with stems defined as first order. For a specific subtrees, Width and Depth are defined as maximal spread in X and Z perpendicular to the main axis; Height is the radial distance from stem origin to the most distant tip. Upper left inset is the 2D Z projection from ImageJ of the original confocal image, centered on the astrocyte for which the centerline tree is presented to illustrate L-Measure descriptors. Upper right inset is the 2D rendering of the 3D space-filing model of the same astrocyte generated by NeuronStudio.

Individual astrocytes fully contained within despeckled confocal image stacks were automatically isolated in 3D from manually entered center points (somata), using Fiji *find connected regions* plugin [39]. Binary images from this process were imported to NeuronStudio [40] for automatic 3D reconstruction and saved in standard swc format. The resulting structure files were analyzed using L-Measure [41] to provide multiple quantitative descriptors of branching and cell morphology. L-Measure is a quantitative morphological measurement tool originally designed for neuronal constructions, but based on general tree variables it is equally suited for analysis of branched structure of astrocytes or other cells. A representative schematic of an astrocyte highlights the elementary topological variables as defined by L-Measure (Fig 2).

### Statistical analysis

Contributions to overall variance were tested using a general linear model (SAS Proc GLM) for the potential effects of overpressure, incubation time, brain hemisphere, image fields (within-slice), slices (within-animal), and interactions. For a given time and treatment condition, the area fraction and intensity data were pooled across the brain hemisphere and image fields since they did not contribute significantly to the variability. Type III sums of squares were used to calculate F values and effects were considered significant at p<0.05. Neither raw intensity data (Lillifors test skewness 0.601, kurtosis 0.561, OpenStat [42]) nor raw area fraction data (Lillifors test skewness 1.459, kurtosis 2.223, OpenStat [42]) were normally distributed. Although ANOVA is tolerant to some deviation from normality, percent differences from mean baseline data were generated, and then normalized by logarithmic transformation. However, raw, percent change from baseline, and log-normalized area fraction and intensity data ANOVAs did not differ in detecting any significant effects or interactions for either area fraction or intensity. Because between-animal variance did not contribute significantly to the overall model, 24 slices from 6 rats were subsequently treated as individual experiments in 2-factor (overpressure x incubation) designs. *Post-hoc* comparisons were done using least-square means. All data are expressed as mean ± standard error (s.e.m).

## Results

### Simulated overpressure wave

The maximum overpressure of the generated stress wave in the test chamber was 60.25 ± 4.99 psi (415.40 ± 34.40 kPa) (Fig 1B), with an overpressure duration of 1–2 milliseconds. The average full width at half maximum pressure indicates the width of the curve. It is defined by the distance between the points on the curve at which pressure reaches half its maximum value and was 362 μs. There was no negative pressure component of the simulated blast wave within 10 ms of gas gun activation. High-speed video documented that the wave transit produced little gross displacement or distortion of slices in the test chamber.

### Quantification of data and statistical analysis

**Fig 3:**
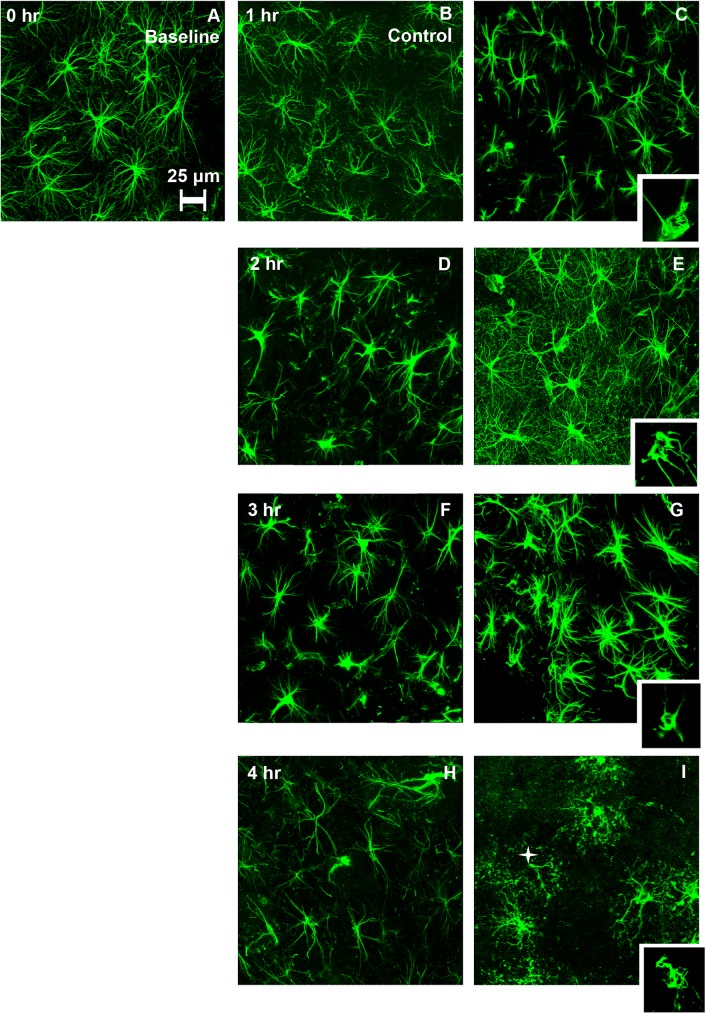
GFAP immunolabeled hippocampal astrocytes from control (left) and overpressure- exposed slices (right) as a function of post-exposure incubation time. Control slices were subjected to similar testing conditions as that of overpressure exposed slices without actual exposure to the overpressure wave. The imaging was restricted to the CA1 subregion of both hippocampi for each slice. (A) At 0 hr. incubation, morphology of the astrocytes is typical to that of a normal brain. (B-C) Astrocytes at 1 hr. incubation time. The morphological features from control (B) and overpressure-exposed (C) look similar with the stellate shape and radial extension. (D-E) Astrocytes at 2 hr. incubation time exhibit qualitative visual differences. overpressure-exposed astrocytes (E) appear hypertrophied and show enhanced GFAP immunofluorescence of the cell bodies along with increased labeling of fine processes when compared to controls (D). (G-F) Astrocytes after 3 hr. incubation. Overpressure-exposed astrocytes (G) showed enhanced GFAP immunofluorescence when compared to control slices (F). (H) Astrocytes from control slices at 4 hr. incubation showed reduced complexity of processes. (I) Clasmatodendritic features were observed in astrocytes from the overpressure-exposed slices when compared to control slices after 4 hr. incubation. Astrocytes showed beaded, presumably disintegrating processes (star). Inset figures present individual confocal planes highlighting abnormal intracellular regions devoid of GFAP immunoreactivity that were never seen in control cells in the cytoplasm along with rounded appearance of somata.

Assessment was conducted over a total of 355 hippocampal CA1 stratum radiatum image fields obtained from 24 slices (6 rats). At 0 hr, morphology of the astrocytes appeared normal with their stellate shape, distinct somata and distinguishable processes (Fig 3A). Qualitative comparison of astrocytes from the control slice images across all other time points (Fig 3B, 3D, 3F & 3H) indicated relatively mild but progressive morphological deterioration over time including loss of finer branches when compared to astrocyte morphology observed at 0 hr incubation time (Fig 3).

### Area fraction of GFAP

**Fig 4:**
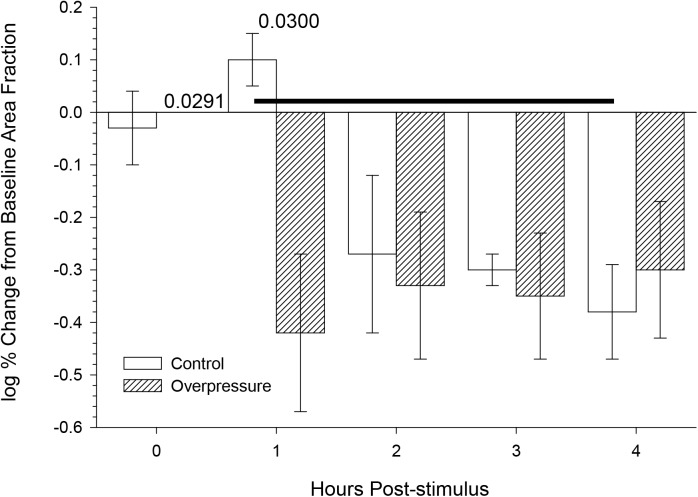
Neither overpressure nor incubation time showed significant main effects on area fraction of GFAP immunoreactivity. Mean ± s.e.m area fraction of GFAP immunoreactivity as a function of incubation time. N within the bars indicates the slices used to collect the data. Each slice contributed to an average of 25 image fields. Main effects (overpressure, incubation) and interactions were tested in a general linear model (SAS Proc GLM) and none were significant. *Post hoc* comparison P values denote significant differences between bar pairs, or bars linked by horizontal line.

Neither overpressure exposure (F(1,17) = 2.60, p = 0.1456) nor incubation time (F(4,17) = 0.3164 had significant effects on the fractional area occupied by GFAP-positive hippocampal astrocytes (Fig 4). Least squares means *post hoc* comparisons between individual conditions showed significantly different changes in area fraction between control slices at 1 and 4 hours, and between control and overpressure-exposed slices at 1 hour.

### Average GFAP intensity

**Fig 5:**
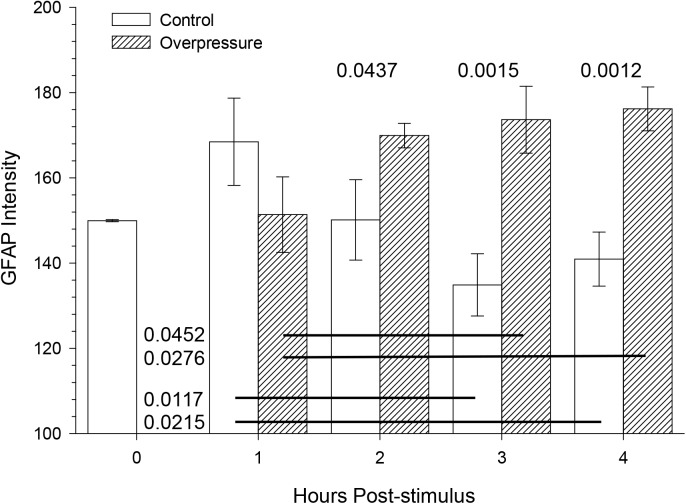
Average GFAP intensity was significantly different between slices exposed to simulated overpressure and controls, over 4 hrs. post testing. Mean ± s.e.m. GFAP immunofluorescence intensity as a function of overpressure and incubation time. N within the bars indicates the slices used to collect the data. Each slice contributed to an average of 25 image fields. Main effects (overpressure, incubation time) and interactions were tested using general linear model (SAS Proc GLM). With the exception of 1 hr. incubation time, the GFAP immunoreactivity in astrocytes was significantly higher than control at all time points. *Post hoc* comparison P values denote significant differences between bar pairs or bars linked by horizontal lines.

The overpressure exposure had a significant effect on the average GFAP intensity (*F(1*,*17) = 14*.*27*, *p = 0*.*0015)* (Fig 5). Incubation time post-testing had no significant effect (*F(4*,*17) = 0*.*421*, *p = 0*.*9280)*. At 2 hours and beyond the intensity values of the average GFAP intensity were higher for overpressure-exposed slices when compared to the control slices. A significant interaction (*F(3*,*17) = 5*.*21*, *p = 0*.*0098)* occurred between the number of hours post exposure and the treatment (overpressure-exposed vs control). Intensity in control slice astrocytes suggested a that mere incubation produced a transient increase followed by a progressive and enduring decrease, in contrast to a delayed and robust increase in overpressure-exposed slices.

### Abnormal intracellular regions devoid of GFAP immunoreactivity

In control slice astrocytes no apparent GFAP-immunonegative intracellular regions in the perikaryal cytoplasm were observed at any time point. In contrast, 9.2 ± 0.8% of astrocytes exhibited the presence of abnormal intracellular regions devoid of GFAP immunoreactivity in the overpressure-exposed samples (Table 1). These intracellular regions were observed at all time points (inset Fig 3C, 3E, 3G and 3I). The fraction of astrocytes with these abnormal intracellular regions devoid of GFAP immunoreactivity as a function of incubation time is reported in Table 1. At 3 hrs post overpressure exposure, 36% of astrocytes displayed multiple GFAP-negative intracellular regions within the cytoplasm (Fig 3G). Individual focal plane images confirm these structures were not artifacts of collapsing images stacks (e.g. overlapping processes in z projections), but true intracellular compartments devoid of immunoreactive GFAP. At 4 hrs post-testing, GFAP-positive processes with a beaded, disintegrated appearance surrounded some of the cell bodies with GFAP-negative intracellular regions (Fig 3I).

**Table 1:** GFAP positive astrocytes with abnormal intracellular regions across incubation time points.

Incubation Time (hr.)	Average Astrocytes/ Image Field		% of Astrocytes with abnormal GFAP-negative intracellular regions (Overpressure-Exposed) *
	Control	Overpressure-Exposed	
1	10.6 ± 0.2	7.8 ± 0.2	9.3 ± 0.7
2	7.5 ± 0.3	8.2 ± 1.3	9.8 ± 0.8
3	11.1 ± 0.3	13.4 ± 1.0	8.0 ± 0.7
4	8.3 ± 0.1	7.7 ± 0.8	9.8 ± 0.5

* No visible GFAP negative intracellular regions were observed in control slices at any incubation time points.

### Time post-injury

Images of CA1 hippocampal astrocytes from control and overpressure-exposed slices revealed clear qualitative differences (Fig 3B through 3I). At 2 hrs post-testing, increased GFAP-positive fine branches were visible in the background (Fig 3E). Whether these branches were connected with astrocytes in the image could not always be determined. At 3 hrs post-exposure, most of the astrocytes in the overpressure-exposed slices displayed increased GFAP immunoreactivity (Fig 3G) with the exception of images obtained from one rat which showed extensive swelling and beading of the distal processes. Extensive disruption of astrocyte morphology with the appearance of beaded processes and GFAP-negative intracellular compartments was a common feature for overpressure-exposed slices at 4 hrs post exposure. However, images obtained from one overpressure-exposed rat at this time point [data not shown] showed extensive enlargement of astrocytes without any beaded appearance but with long main processes that had periodic breaks along with large vacuoles and watery nucleus as described by Ryu et al [43].

### Quantitative morphological assessment

Morphometric data for 239 astrocytes (150 from overpressure-exposed slices) were analyzed to identify effects of shock, incubation time, and interactions. Main effects and interactions of slice incubation time and simulated overpressure shock exposure were detected for many of the quantitative descriptors returned by L-Measure (Table 2). As many of these correlate (e.g. number of branches and number of branch tips), effects and interactions are shared across multiple properties. Some variables are useful as indices of individual branch properties as well as overall cell structure, but some are only meaningful for individual arbors (e.g. width) or entire cells (e.g. number of stems).

**Table 2:** Significant effects of blast-overpressure (B), incubation time (T), and interactions (I) for morphological properties of GFAP+ astrocytes.

Metric	Total_Sum	Average
Soma_Surface *	T, I	
N_stems	T	
N_bifs	B, T, I	
N_branch	B, T, I	
N_tips	B, T, I	
Width		B, T
Height		B, T, I
Depth		T, I
Diameter		B, T, I
Diameter_pow	B, T, I	T, I
Length	B, T, I	B, T
Surface	B, T	B, T, I
SectionArea	T, I	B, T, I
Volume	B, T, I	T
EucDistance	B, T, I	B, T, I
PathDistance	B, T,I	B, T, I
Branch_Order	B, T, I	B, T
Terminal_degree	B, T, I	I
TerminalSegment	B, T, I	
Taper_1	T	T, I
Taper_2	T	T, I
Branch_pathlength	B, T, I	T, I
Contraction	B, T, I	T, I
Fragmentation		B, T, I
Daughter_Ratio		T, I
Parent_Daughter_Ratio		T
Partition_asymmetry	T, I	B, T, I
Pk		B, T, I
Pk_classic		B
Bif_ampl_remote		T
Bif_tilt_local		B, T
Bif_tilt_remote		B, T, I
Bif_torque_local		T
Last_parent_diam	B, T, I	T, I
Diam_threshold	B, T, I	T, I
HillmanThreshold	T	B, T, I
Fractal_Dim	B, T, I	T, I

**Fig 6:**
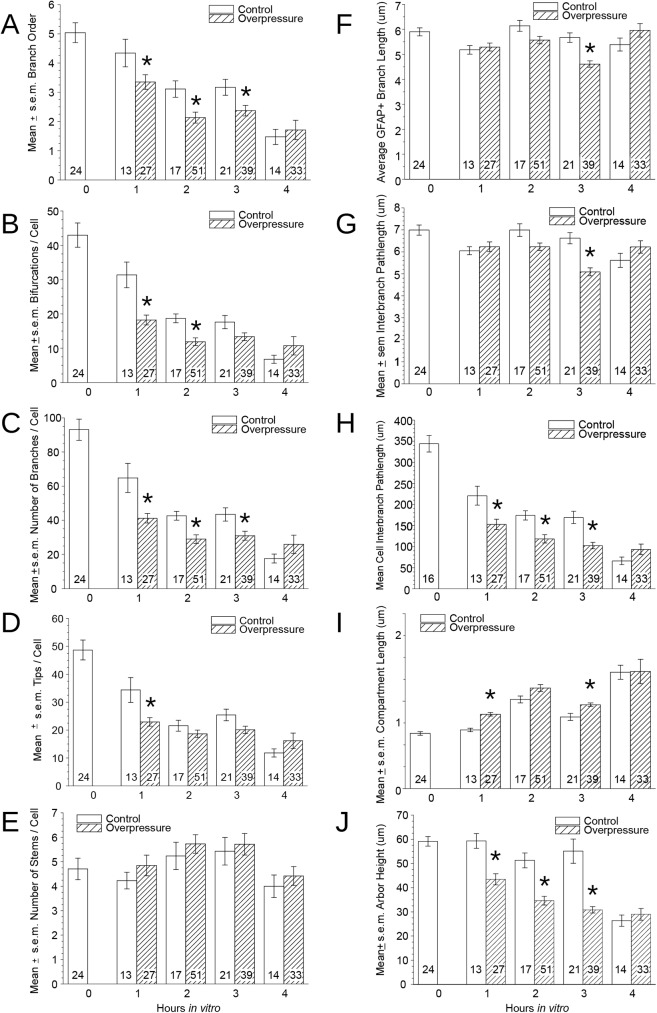
Morphometric properties of GFAP-immunoreactive astrocytes reveal effects of and interactions between brain slice incubation time and overpressure exposure. (A) Average branch order is reduced by 1 hr post-testing but not in control slices from a sample of GFAP+ astrocytes selected for isolation (non-overlapping) and full containment in confocal image stacks. A relative reduction in branch order defining a overpressure effect was evident in overpressure slice cells until 3 hours, after which cells in both groups exhibited few branches beyond primary.(B, C, D) Blast and incubation time have coordinated effects on numbers of branch points, branches, and branch tips. (E) Branches emerging from cell bodies are not affected by overpressure at any time point, or incubation before 4 hrs. (F) Individual branch length comprised of stems and tips was not affected by overpressure or incubation time. (G) Difference in inter-branch segments was not significant between the two treatment groups. (H) Reduction in total length of branches per cell was substantial and progressive with overpressure and time, with essentially total elimination in the 4 hr overpressure sample. (I) An accompanying increase in average compartment lengths suggests either straightening of branches or loss of tortuous processes [44]. A small but significant effect of overpressure is observed at early time points. (J) Average arbor height (distance from soma to most distal tip) was reduced early and progressively by shock, compared to relative stability in controls before 4 hrs. Asterisks reflect *p < 0*.*05* in *post-hoc* comparisons between adjacent conditions; significant main effects and interactions are summarized in Table 2.

Formal variable definitions not included in Fig 6 can be found at http://cng.gmu.edu:8080/Lm/help/index.htm [41]. For a given variable, Total_Sum is defined as the sum of all the values for a chosen cell, and Average is defined as the mean of all the values for the particular cell. The Surface, Section Area, and Volume variables exclude somatic compartments and thus reflect characteristics of GFAP+ branches only.

* Because NeuronStudio returns a single spherical compartment for somata, Soma_Surface values are not accurate.

Branch order, a useful overall measure of arborization, was reduced 1 hr after overpressure-exposure but not in incubation control slices (Fig 6A). The numbers of branch points, branches, and tips were coordinately affected by incubation time and overpressure, and interactions were observed such that the proportional temporal reduction was more pronounced in control slices (Fig 6B through 6D). Changes associated with overpressure exposure were rapid and robust. At 1 hr overpressure-exposed astrocytes had over 40% reduction in branch points. Preservation of numbers of stems emerging from somata (until 4 hrs) (Fig 6E) indicates that distal branches rather than entire arbors were lost.

Despite loss of branches the average length of individual branches did not change (Fig 6F and 6G). Total process length was affected by overpressure and by time individually but the interaction was not significant. GFAP+ process length per cell decreased over 1/3 during the first hour of incubation, and overpressure-exposure compounded this reduction (Fig 6H). By 4 hrs after overpressure few GFAP+ processes were present (<1% original), but control slice astrocytes maintained 17% of their original process length. Blast-overpressure also had a small but significant early effect on process compartment lengths that post-overpressure incubation did not (Fig 6I), although this metric did show a time-related increase in both conditions.

Even with some loss of tips, the average distance from the soma to most distal GFAP+ process tip of individual arbors did not change in control slice astrocytes until 4 hrs after injury (Fig 6J). This contrasts with overpressure, which shortened entire arbors early and progressively. Taper, contraction, and number of stems per cell did not show significant effects of incubation alone but were sensitive to overpressure-exposure.

## Discussion

Blast wave characteristics (magnitude, velocity, direction) determine the specific local forces that are exerted on tissue structures [34].The respective contribution of these different factors to blast injuries is currently unclear. The combination of an advanced blast-overpressure simulation technology with a controlled experimental environment for testing the response of live, embedded cells provides a platform for analyzing specific effects isolated from external confounds. Its use in the present study provides insight on overpressure injury that is otherwise difficult to isolate. Improvements in the test system designs resulted in a controlled lower overpressure profile (~60psi, 1-2ms) and presumably lower more realistic strains compared to higher pressures (~1500 psi, 5 ms) with a substantial underpressure component utilized in our previous study. Although the current model does not account for wave reflections, the magnitude of the pressure used in this study was slightly higher but within the same order of magnitude as reported by other studies that have looked at intracranial pressure in rat models [~ 50 psi for 20 psi exposure; [35] ~ 6 psi for 5 psi exposure; [36] <1 psi, for 1.45 psi exposure [45]].

Analogous to our previous studies of blast effects on neurons [30], this approach was able to distinguish overpressure-induced changes in astrocyte characteristics from those intrinsic to the acute slice preparation [44]. Evolving astrocyte pathology emerges during the initial hours after exposure to overpressure alone at magnitudes and durations comparable to real blast waves present inside the head. Increased GFAP intensity was observed in overpressure-tested tissue samples as early as 2 hrs post testing (Fig 5), and morphological effects were observed at 1 hr when compared to time matched controls. Current results align with the recent evidence of elevation of serum GFAP breakdown products observed in established TBI rodent models which is thought to be associated with astrocyte damage or cell death after brain injury along with compromised blood brain barrier [46].The progressive nature of the injury is indicated by significant interaction between treatment (overpressure-exposed vs. control) and time post-exposure. The data suggest that astrocyte injury is at least concurrent with and in fact precedes the time when we could detect neuronal injury at 6 hours post injury in a similar acute live slice model [30], and that compensatory responses to injury can be detected almost immediately after overpressure exposure.

These blast effects are far more rapid that what is generally considered the acute phase of reactive astrocytosis [47]. Reactive astroglia have been observed one day after blast wave exposure [13, 14, 48] and reported to persist for 30 days after exposure [1] to higher pressures (17–52 psi (117.21–358.52 kPa), 3–10 ms). Our results complement a recent report describing changes in GFAP immunofluorescence, and dramatic fine process loss in astrocytes, after 1 hr incubations of slices taken from mice expressing green fluorescent protein in astrocytes [44]. Although we did not observe the same GFAP fluorescence increase in rat slices with incubation alone, the degree and timing of progressive fine process loss in mouse slices corresponds well with the loss of branch tips measured in our study. Whether these early effects represent initial components of traditional reactive astrocytosis or separate processes, they further establish that astrocytes respond to injury more quickly than is generally appreciated. Markers for apoptosis characterized by nuclear condensation and fragmentation, caspase activation and membrane blebbing can provide useful insight into the biochemically and morphologically distinct blast-induced astrocyte death mechanisms. Evidence of classical apoptosis in a subset of cerebral astrocytes as early as 12 hours after ischemia allude to a blast model with reduced background cell death not feasible with the slice model used in this study [49].

The observed increase in GFAP immunofluorescence intensity could stem from either increased expression of GFAP or an intracellular increase in epitope accessibility. The earliest changes could reflect the dynamic balance between cytoskeletal (polymerized) and soluble, monomeric GFAP [50–52]. Although reorganization of GFAP subunits leading to increased epitope availability due to edema [53], glial damage [54], or transient acidification [55] could affect astrocytes in both the treatment groups, compromised cell membrane due to blast exposure likely supplemented antibody accessibility [2]. Temporal constraints on increasing protein expression make it more probable that the later occurring increases in immunoreactivity could involve true upregulation. Proliferation would not be likely within the survival duration tested [8], but the observed early events could be involved in activating cell division with effects detectable later.

Qualitatively, two distinct astrocyte injury profiles were identified: (a) a process similar in one respect to conventional astrogliosis, with an increase in GFAP immunoreactivity in GFAP-positive cells, and (b) a process comparable to clasmatodendrosis, an acute autolytic phenomenon, characterized by voids in cytoplasm in conjunction with rounded somata and disintegrating processes. The latter has not been previously associated with blast-induced injury, and neither has been previously demonstrated as acute responses reflecting distinct astrocyte vulnerability. Described by Cajal in the early 1900’s [56], clasmatodendrosis was only observed in blast-exposed slices and was present at all time points. It did however appear to follow a progressive path with initial exhibition of GFAP-immunonegative intracellular regions appearing alongside rounded somata before the appearance of beaded and disintegrated processes. This irreversible degenerative morphology of astrocytes has been observed in brain samples linked to various disease states [Alzheimer’s disease in conjunction with cerebrovascular disorder [57]; epilepsy [43, 58]]. This astrocyte pathology has not been previously associated with blast induced neurotrauma, but clasmatodendritic astrocytes were present in human cerebral cortices as early as 1 hr after non-blast-induced traumatic brain injury [59]. Higher strain rates concurrent with blast exposure have been shown to induce blebbing in a subset of neurons due to strain stiffening of the cytoskeletal proteins [60]. Whether blast-induced-high-strain- rates or energy failure and acidosis previously reasoned as causal factors for clasmatodendrosis [59], accounted for observations in this study is not clear.

With fragmentation preventing 3D reconstruction of clasmatodendritic astrocytes, our quantitative morphometry sample is biased toward less severely affected cells. The fact that GFAP does not reveal the entire astrocyte morphology [47] also limits a full appreciation for changes in cell structure. Nonetheless, this approach demonstrated that relatively rapid, substantial, and apparently independent changes in arborization could be attributed to overpressure-exposure and incubation time. In fact, even with selection of less affected astrocytes, the objective definition of process connectivity applied by the automatic 3D tracing algorithm proved to be far more sensitive at detecting morphological effects than qualitative comparisons represented in Fig 3.

In addition to significant interactions between slice incubation time and blast-overpressure on many properties, overpressure effects were detected in the absence of incubation effects for number of stems per cell, and for branch taper. Thus overpressure-exposure is not simply additive to the progressive decline of viability inherent to the acute slice preparation [32], but invokes distinct pathological mechanisms with rapid onset. An additional clue to potential differences in the mechanisms may be the coordinated loss of distal branches (i.e., both tips) necessary for overpressure to reduce arbor height (Fig 6J) which is absent from incubation despite also being associated with a significant (though smaller) loss of tips. That height did not increase also indicates that affected cells did not expand their original spatial domains.

Some limitations of this current study should be acknowledged. Although the use of acute slice model allowed for study for viable astrocytes in native tissue architecture, experimental treatment effects can only be detected in the context of gradual decline in tissue viability. Continued evolution of treatment effects beyond the duration of viable tissue is not possible. Typical of acute slice preparations, analyses are limited to a structure where local relative cytological homogeneity favors comparisons across animals and conditions. Considering this understanding we designed the experiment to focus on the hippocampus and specifically the CA1 sub region which has been previously shown to be prone to blast wave transmission. However, regional variation of the heterogeneous astrocyte susceptibility (for example radial vs fibrous) is important and will be focus of our future studies. The simulated overpressure intensities were based on real explosive blasts and TBI studies; however experiments designed with lower pressures magnitudes to stimulate mild blast conditions with further modification of the test system will help elucidate the specific role of overpressure induced injury compared to a simulated blast profile. Coordinated analyses of the many cell types found in brain may provide better insight into interactions that define injury response.

Human cortical astrocytes have been reported to encompass 2 million synaptic terminals [61] and to exhibit physiological complexity and diversity comparable to neurons. Their substantially larger size and long processes in human cortex predict even higher vulnerability to high-strain rate-induced mechanical injury than what we observed in rat brain tissue and proportionately greater consequences for neurons wholly or partly within their spatial domains. Even temporary compromise of lactate delivery from astrocyte, or uptake of extracellular potassium or glutamate, has the potential to impose lasting and distributed effects on neurons and emergent functions. The data are entirely consistent with the hypothesis that astrocyte injury could affect multineuronal pathology sufficient for blast-related dysfunction at a scale below current imaging resolutions.
